# *Cordyceps sinensis* (CS) Alleviates Chronic Obstructive Pulmonary Disease Symptoms (COPD) by Targeting Inflammation, Apoptosis, and Oxidative Stress Through Ingredient–Gene–Disease Interaction

**DOI:** 10.1002/iid3.70306

**Published:** 2025-12-15

**Authors:** Zirui Zang, Yuhan Kong, Qi Kong

**Affiliations:** ^1^ Capital Medical University Beijing China; ^2^ Capital Normal University Beijing China; ^3^ Institute of Laboratory Animal Sciences, Chinese Academy of Medical Sciences (CAMS) and Comparative Medicine Center Peking Union Medical College (PUMC) Beijing China; ^4^ Key Laboratory of Human Disease Comparative Medicine Chinese Ministry of Health Beijing China; ^5^ Beijing Key Laboratory for Animal Models of Emerging and Remerging Infectious Diseases Beijing China

**Keywords:** apoptosis, autophagy, chronic obstructive pulmonary disease, *Cordyceps sinensis*, inflammation, oxidative stress

## Abstract

**Objective:**

*Cordyceps sinensis* (CS) is a fungus that parasitizes the larvae and corpses of Batmadaceae insects. CS is used as a traditional Chinese medicine and has shown promising clinical efficacy in the treatment of chronic obstructive pulmonary disease (COPD). This study aimed to identify potential targets of CS in the treatment of COPD and to analyse the related biological processes and signalling pathways.

**Methods:**

Through the use of network pharmacological tools, Gene Ontology (GO), and Kyoto Encyclopedia of Genes and Genomes (KEGG) pathway analyses, as well as corroborating data from Gene Expression Omnibus (GEO) and the literature, potential targets for CS treatment of COPD were identified and analysed.

**Results:**

These results suggest that CS alleviates COPD symptoms with key biomarkers such as interleukins (ILs), tumour necrosis factor (TNF), and reactive oxygen species (ROS). The primary active constituents of CS comprise Cordycepin, d‐mannitol, Ergosterol, Cordyceps polysaccharides, and others. During the pathogenesis of COPD, CS exerts modulates effects on various proteins and signalling pathways, influencing gene expression patterns such as poly ADP‐ribose polymerase 1 (PARP1), phosphodiesterase 4A (PDE4A), phosphodiesterase 4B (PDE4B), phosphodiesterase 4C (PDE4C), phosphodiesterase 4D (PDE4D), prostaglandin D2 receptor 2 (PTGDR2), heme oxygenase 1 (HMOX1), and matrix metallopeptidase 1 (MMP1).

**Conclusion:**

CS alleviates COPD symptoms by suppressing inflammation, apoptosis, and oxidative stress, suggesting novel therapeutic strategies.

## Graphical abstract

Our findings indicate that Cordyceps Sinensis (CS) alleviates COPD symptoms by inhibiting inflammation and oxidative stress, with key biomarkers such as interleukins (ILs), tumor necrosis factor (TNF), and reactive oxygen species (ROS) identified. Activating autophagy serves as a protective mechanism against COPD development induced by oxidative stress. This study suggests that CS may alleviate COPD symptoms by targeting inflammation, apoptosis and oxidative stress, offering a new perspective for developing more effective treatment methods for COPD.

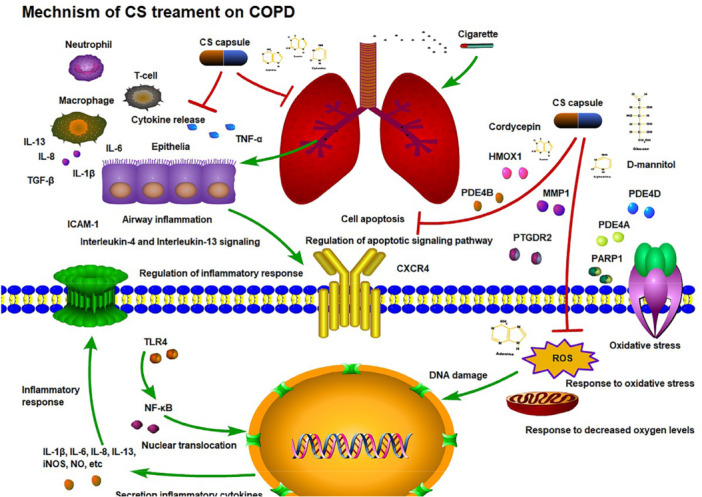

## 1 Introduction

Chronic obstructive pulmonary disease (COPD) is a severe chronic respiratory disease characterized by a high incidence, rapid progression, high disability rate, and high mortality worldwide. In China, the burden of COPD is particularly severe, with an estimated 100 million patients accounting for approximately 25% of all cases worldwide [1]. Inflammation, the immune response, cell apoptosis, and oxidative stress are recognized as key pathological mechanisms in COPD.

Traditional Chinese medicine (TCM) has a long history of treating COPD, offering beneficial effects through multiple targets and pathways. *Cordyceps sinensis* (CS) has been extensively studied for its safety and efficacy in COPD treatment [2]. CS and *Cordyceps militaris* (CM) are commonly utilized in various Chinese patent drugs or products, such as Bailing capsules, Qingfei capsules, Guben Qiangshen capsules, CS Wuwei granules, and Jinshuibao capsules [3].

The composition of CS includes 10.84% moisture, 25.32% crude protein, 18.55% crude fibre, 28.9% carbohydrates, 4.1% ash, and 8.4% fat. Pharmacological investigations have revealed that CS contains cordycepin (0.5%), d‐mannitol (cordycepic acid, 7%), polysaccharides (6.62 g/100 g), ergosterol (2.6 mg/g–3.6 mg/g), glycosides, various amino acids, selenium, zinc, strontium, and other elements [4]. CS and its active ingredients have numerous pharmacological effects, such as anti‐inflammatory, antioxidant, antitumour, antiapoptotic, and immunomodulatory effects [5].

However, the underlying molecular mechanisms remain incompletely understood [6]. Animal studies have highlighted the notable anti‐inflammatory properties of CS and its components, effectively reducing airway structural alterations. Nevertheless, the precise mechanisms underlying the ability of CS to alleviate bronchial inflammation and lung tissue remodelling remain unclear. Despite its common use as a complementary treatment for COPD, the precise pharmacological and therapeutic targets of CS in this process need further exploration.

This study aimed to identify the potential targets of CS in the treatment of COPD and elucidate the related biological processes and signalling pathways. Identifying specific targets and pathways involved in CS's therapeutic effects could pave the way for personalized treatment approaches tailored to individual patient needs. Knowledge of CS's molecular targets could facilitate the development of combination therapies that synergistically enhance treatment outcomes. These findings could lead to a deeper understanding of CS's mode of action.

The relevance and novelty of this study lie in its focus on the molecular level analysis of CS's therapeutic effects on COPD. By using network pharmacological tools, GO, and KEGG pathway analyses, as well as corroborating data from GEO and the literature, we aim to provide a comprehensive view of CS's mode of action. The findings of this study have the potential to significantly advance our understanding of COPD pathology and the therapeutic mechanisms of CS, ultimately contributing to improved patient outcomes and quality of life.

The intricate relationships among inflammation, the immune response, cell apoptosis, and oxidative stress continue to intrigue doctors and researchers alike. Network pharmacology and bioinformatics analyses offer a plethora of tools and methodologies to elucidate the active ingredients of CS, the co‐targeted proteins in CS‐COPD, the intricate interactions between ingredient–protein complexes, and the pivotal roles of various pathways. In our study, we endeavoured to decipher the targets of CS in COPD through rigorous network pharmacology analyses, which were further validated by molecular docking simulations between CS ingredients and targeted proteins and corroborated by gene expression data.

## 2 Materials and Methods

### 2.1 CS Ingredient‐Targeted Genes and Enrichment Analyses

BATMAN‐TCM (http://bionet.ncpsb.org.cn/batman-tcm/#/home) was used in this study to explore the molecular mechanisms of traditional Chinese medicine (TCM) by predicting the potential targets of CS ingredients [7]. Only candidate targets with scores equal to or greater than 0.84 (likelihood ratio (LR) = 80.88) were included in the subsequent analyses. Enrichment analysis was conducted to evaluate Gene Ontology (GO) terms, Kyoto Encyclopedia of Genes and Genomes (KEGG) pathways, and Therapeutic Target Database (TTD) diseases associated with the identified target proteins. The top 10 most significantly enriched KEGG pathways/GO terms/TTD diseases with adjusted P values less than 0.05 were selected for further investigation.

### 2.2 CS Ingredient Network Pharmacology Analyses

HERB (http://herb.ac.cn) was used to gather detailed information on CS through high‐throughput experimental data (GSE24191) and reference‐guided TCM database searches [8]. We also used the STITCH database (http://stitch.embl.de/) to explore known and predicted interactions of CS ingredients and potential targeted proteins on the basis of evidence derived from experiments, databases, and the literature [9].

### 2.3 CS‐COPD Co‐Targeted Genes Known and Prediction

GeneCards (https://www.genecards.org/) is a comprehensive human gene database that provides detailed genomic information on known and predicted human genes [10]. DisGeNET (v7.0, https://www.disgenet.org/) contains 1,134,942 gene–disease associations (GDAs), including 21,671 genes and 30,170 diseases, disorders, and traits. The CDC Public Health Genomics and Precision Health Knowledge Base (PHGKB, https://phgkb.cdc.gov/) is an online database that is continuously updated and searchable and focuses on translating genomics and precision health discoveries into health care and disease prevention. In our study, we identified COPD‐related genes by searching for the keywords ‘Pulmonary Disease, Chronic Obstructive’ in GeneCards, DisGeNET, and PHGKB separately.

The STRING database (version 12.0, https://cn.string-db.org/) was used to explore protein–protein interactions (PPIs), including known and predicted associations [11]. Our analysis aimed to investigate the impact of CS treatment on COPD‐related genes, emphasizing PPIs, and the enrichment of GO and KEGG pathways. Additionally, we used the STRING database to identify known and predicted PPIs with 219 DEGs from GSE126157 and to analyse the effects of cordycepin treatment on mouse monocytic macrophage leukemia cell Line (RAW 264.7 cells) with lipopolysaccharides (LPS) induced inflammation [12].

### 2.4 Analyses of the CS‐COPD Ingredient–Gene–Disease Network

The CS‐COPD ingredient–gene–disease network was analysed via Metascape (version 3.5, https://Metascape.org/), a gene list annotation platform that integrates various functions, such as functional enrichment, interactome analysis, gene annotation, and membership within a single portal [13]. The effect of cordycepin on COPD‐related genes was explored through batch mode analysis of 219 differentially expressed genes (DEGs) from GSE126157, which involved treating eight samples of RAW 264.7 macrophages with 1 µg/ml LPS and incubating them for 60 min in 20 µM (20 μmol/L) cordycepin or dimethyl sulfoxide (DMSO) [14].

### 2.5 Transcriptomic Data Analysis of the Effects of the CS Ingredients on Mouse Cells

Transcriptomic data analysis of the effects of CS in human and mouse cells included the retrieval of transcriptome microarray data from the GEO database (gene expression omnibus, https://www.ncbi.nlm.nih.gov/geo/), specifically GSE126157 (cordycepin on murine macrophage‐like cells) [12] and GSE24191 (dendritic cells treated with CS) [15]. The statistical methods used analysis of variance (ANOVA) in Figure 3 and 5. These DEGs were selected based on their statistical significance (*p* < 0.05) and potential relevance to the anti‐inflammatory effects of cordycepin. The results were visualized using GraphPad Prism 9.0 software, as indicated in the figures.

To investigate the anti‐inflammatory properties of cordycepin, GSE126157 utilized RAW 264.7 cells, which were stimulated with 1 μg/mL LPS for 10 min and then treated with 20 μM (20 μmol/L) cordycepin [14]. Transcriptomic data were analyzed using GEO2R, and statistical comparisons were performed using ANOVA. DEGs were identified based on adjusted *p*‐values and fold changes.

### 2.6 Molecular Docking Analysis of CS Ingredients and Targeted Genes

We utilized the SwissDock online tool (https://www.swissdock.ch/) to perform molecular docking analysis of the CS ingredients and their targets [16]. The coordinates of the targets were retrieved from the protein data bank (PDB, https://www.rcsb.org/). The molecular structures of the CS ingredients were retrieved from the PubChem compound database (https://pubchem.ncbi.nlm.nih.gov/). Before docking analysis, all protein and molecular files were converted to the PDB partial charge and atom type format (PDBQT) format, excluding water molecules and including polar hydrogen atoms [17].

## 3 Results

### 3.1 Enrichment Analyses of Predicted Candidate Targets and CS Ingredients

**Figure 1:**
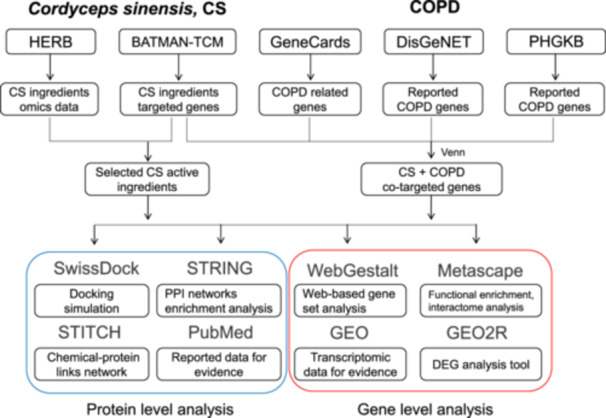
The step‐by‐step pipeline of the data analysis and network pharmacology approach in this study.

The study design is depicted in Figure 1, which shows a detailed pipeline of the data analysis and network pharmacology approach. BATMAN‐TCM 2.0 identified 240 ingredients of CS, with two ingredients having no match with the predicted target proteins. The system identifies candidate target proteins with confidence scores ≥ 0.84 and druggable scores ≥ 0.1. In total, 123 ingredients were associated with 4238 predicted target proteins, highlighting the active components of CS.

CS contains various ingredients, such as cordycepin, d‐mannitol, cordyceps polysaccharides, sterols, amino acids, fatty acids, carbohydrates, and vitamin B12. Cordyceps polysaccharides mainly consist of galactomannan, which is composed of d‐galactose and d‐glucoside at a 1:1 molar ratio. Enrichment analyses of the predicted candidate targets were performed based on scores of 20 or higher for each TCM ingredient query.

Table 1 presents the top 10 significantly enriched functional terms (GO term, KEGG pathway, and TTD disease) with adjusted *p* values < 0.05. The most enriched GO terms for the predicted candidate targets included Homeostatic Process, Transport, Response to Stress, Cell–Cell Signalling, Lipid Metabolic Process, Cell Death, Signal Transduction, and Oxidoreductase Activity.

**Table 1:** Gene Ontology (GO) enrichment analyses were conducted on the predicted top 10 candidate targets for CS using BATMAN‐TCM 2.0.

GO id	Description	*p*‐value	Benjamini‐value	Gene number	Enrich ratio
GO:0042592	Homeostatic process	1.27e^−75^	1.74e−^73^	208	4.1
GO:0006810	Transport	3.86e^−75^	2.64e^−73^	395	2.3
GO:0006950	Response to stress	1.2e^−68^	5.46e^−67^	334	2.5
GO:0007267	Cell–cell signaling	7.57e^−63^	2.07e^−61^	192	3.8
GO:0006629	Lipid metabolic process	1.8e^−52^	3.53e^−51^	181	3.4
GO:0003013	Circulatory system process	1.44e^−46^	1.97e^−45^	87	6.3
GO:0008219	Cell death	1.91e^−37^	2.27e^−36^	200	2.5
GO:0007165	Signal transduction	1.99e^−37^	2.27e^−36^	385	1.7
GO:0016491	Oxidoreductase activity	1.59e^−36^	1.67e^−35^	130	3.4
GO:0050877	Neurological system process	5.21e^−35^	5.1e^−34^	191	2.5

*Note: p*‐value < 0.01; Benjamini‐value < 0.01; Enrich Ratio> 1.5.

This study demonstrated that CS‐targeted genes affect multiple KEGG pathways, as outlined in Table 2. Specifically, the Calcium signalling pathway, cGMP‐PKG signalling pathway, PPAR signalling pathway, and Adipocytokine signalling pathway are linked to the anti‐inflammatory and antioxidant properties of CS.

**Table 2:** KEGG enrichment analyses of the top 10 predicted candidate targets for CS using BATMAN‐TCM 2.0.

Pathway classification	KEGG pathway	*p*‐value	Benjamini‐value	Gene number	Enrich ratio
Signaling molecules and interaction	hsa04080 Neuroactive ligand‐receptor interaction	1.38e^−36^	3.08e^−34^	102	3.8
Signal transduction	hsa04020 Calcium signaling pathway	8.36e^−22^	9.32e^−20^	64	3.6
Signal transduction	hsa04022 cGMP ‐ PKG signaling pathway	9.60e^−16^	4.28e^−14^	53	3.3
Nervous system	hsa04726 Serotonergic synapse	1.99e^−15^	7.40e^−14^	42	3.8
Digestive system	hsa04976 Bile secretion	3.06e^−11^	6.82e^−10^	28	4
Nervous system	hsa04723 Retrograde endocannabinoid signaling	1.72e^−10^	3.49e^−09^	33	3.3
Nervous system	hsa04727 GABAergic synapse	3.83e^−08^	6.57e^−07^	27	3.1
Endocrine system	hsa03320 PPAR signaling pathway	5.65e^−08^	9.00e^−07^	23	3.4
Nucleotide metabolism	hsa00230 Purine metabolism	2.03e^−07^	2.83e^−06^	39	2.3
Endocrine system	hsa04920 Adipocytokine signaling pathway	1.56e^−06^	1.74e^−05^	21	3.1

*Note: p*‐value < 0.01; Benjamini‐value < 0.01; Enrich Ratio> 1.5.

In COPD, CS influences genes such as PARP1, PDE4A, PDE4B, PDE4C, PDE4D, PTGDR2 (*p* = 4.61e^‐03^, TTD), HMOX1, and MMP1 (*p* = 3.65e^‐02^, OMIM). The PDE4A protein, which is part of the PDE4 subfamily within the cyclic nucleotide phosphodiesterase (PDE) family, is crucial for regulating cellular cAMP levels and is involved in various physiological processes (Table 3). Using BATMAN‐TCM 2.0, GeneCards, and DisGeNET, we examined co‐targeted genes in CS‐COPD, along with predicted scores and key ingredients. These findings indicate that several ingredients play a role in the treatment of COPD with CS.

**Table 3:** Analysis results of enrichment of co‐targeted genes in CS‐COPD and related key ingredients, including Cordycepin, d‐Mannitol, Ergotamine, etc.

Genes	Gene name	Gene Cards score	DisGe NET pLI^a^	Ingredients number	Key ingredients in BATMAN‐TCM 2.0 (scores)
PARP1	Poly(ADP‐Ribose) Polymerase 1	30.61	3.30e^‐04^	20	Caffeine (278.000), Nicotinamide, Nicotinic Acid Hydrazide (122.780), Arachidic Acid (80.880), Riboflavin (36.690), Adenine (25.860), Palmitic Acid (36.690), Adenosine (39.420)
PTGDR2	Prostaglandin D2 Receptor 2	13.55	—	20	Adenosine (183.000), Arachidonic Acid (140.500), Oleic Acid (48.000), Linoleic Acid (48.000), Isolinoleic Acid (22.307)
PDE4A	Phosphodiesterase 4A	38.84	0.98	23	2′‐Deoxyadenosine (373.000), d‐Mannitol (36.690), Adenine (25.860), Uric Acid (15.370), Adenosine (25.860)
PDE4B	Phosphodiesterase 4B	12.17	0.47	37	Adenine (373.000), Adenosine (122.780), Cordycepin (48.000), 2′‐Deoxyadenosine (48.000), Inosine (48.000), d‐Mannitol (36.690), Guanosine(15.370), 5,6‐Epoxyergosterol (15.370)
PDE4C	Phosphodiesterase 4C	4.04	ND^b^	19	d‐Mannitol (36.690), Uric Acid (15.370), Adenosine (25.860)
PDE4D	Phosphodiesterase 4D	25.27	1	40	Adenine (373.000), Adenosine (122.780), Cordycepin (48.000), 2′‐Deoxyadenosine (48.000), Inosine (48.000), Ergotamine (39.420), d‐Mannitol (36.690), Ergotaminine (22.370), Guanosine (15.370), 5,6‐Epoxyergosterol (15.370)
HMOX1	Heme Oxygenase 1	113.04	9.70e^‐03^	21	Cordycepin (15.370), Riboflavin (26.370), Guanosine (23.000), Adenosine (23.000), Inosine (15.370), Higenamine (15.370), 2′‐Deoxyguanosine(15.370)
MMP1	matrix metallopeptidase 1	116.24	7.70e^‐18^	3	Ergotamine (13.260), Caffeine (13.260), Berberine (known)

a DisGeNET pLI is defined as the probability of being loss‐of‐function intolerant. LoF tolerant genes will have low pLI values (≤ 0.1).

^b^ ND, not detected.

### 3.2 CS‐COPD Co‐Targeted Known Genes and Prediction of Protein–Protein Interactions

Using the keywords ‘Pulmonary Disease, Chronic Obstructive’, a total of 8722 genes were identified in GeneCards, 1428 genes were identified in DisGeNET, and 787 genes were identified in PHGKB (v9.0). There are 211 genes that are co‐targeted for COPD treatment across BATMAN‐TCM 2.0, GeneCards, DisGeNET, and PHGKB. Further analysis included GO enrichment of CS‐related genes from high‐throughput experiments via the HERB database. The GO biological process function of CS was enriched in three categories: sugar biosynthetic process, apoptotic signalling pathway, and oxidative stress pathway. The GO biological process function of cordycepin was enriched in three categories. The first category includes the regulation of cell–cell adhesion, such as the regulation of leukocyte cell–cell adhesion, the regulation of cell–cell adhesion, and leukocyte cell–cell adhesion. The second category encompasses regulation of the inflammatory response, consisting of regulation of the inflammatory response, positive regulation of T‐cell activation, regulation of T‐cell activation, and T‐cell activation.

**Figure 2:**
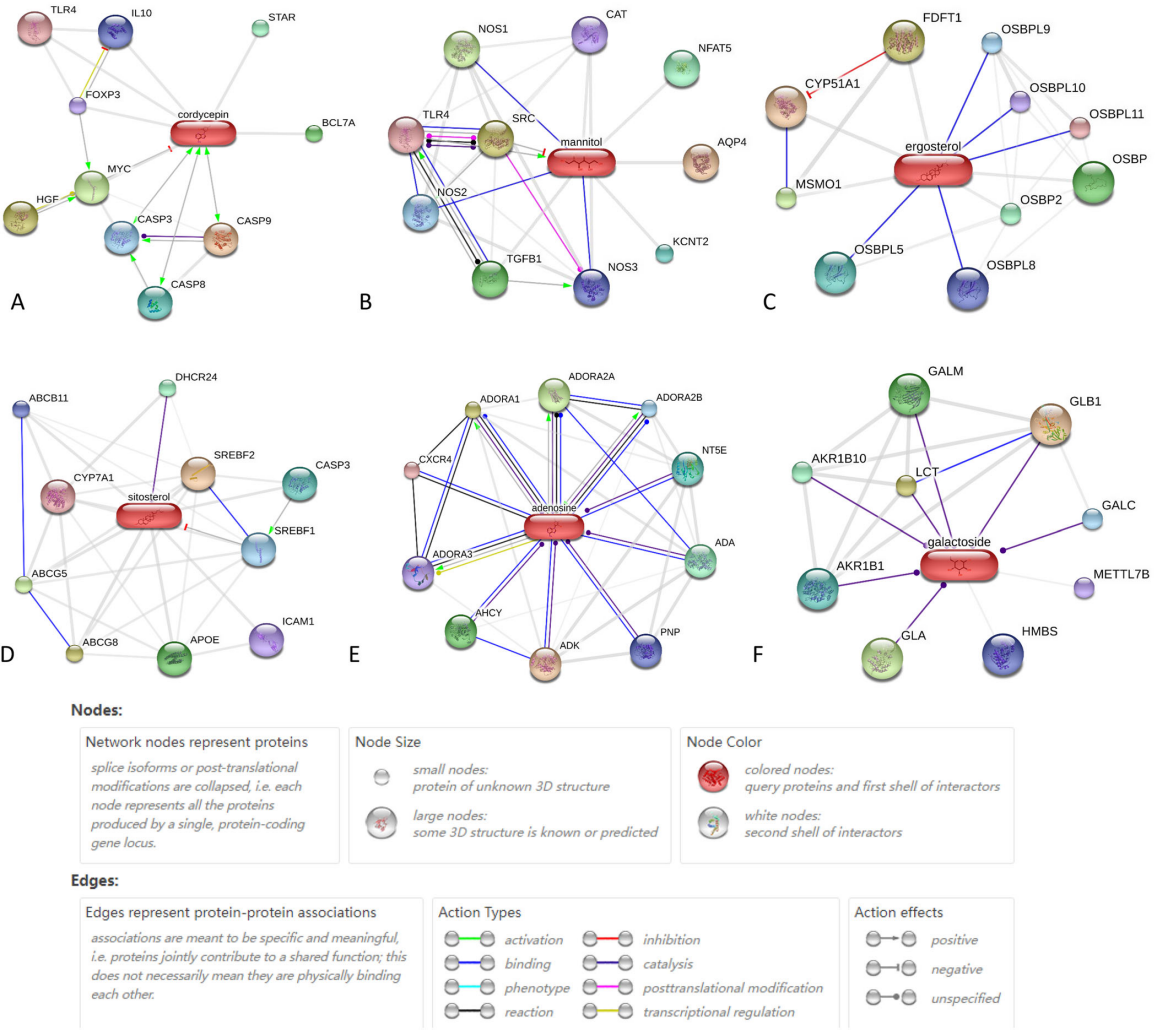
Predicted primary active ingredients of CS obtained from the STITCH database in the actions view. (A) cordycepin; (B) d‐mannitol (cordycepic acid); (C) ergosterol; (D) β‐sitosterol; (E) adenosine; (F) d‐glucoside (cordyceps polysaccharide). The modes of interaction between the chemicals and proteins are highlighted in distinct colors.

Figure 2 presents the key active ingredients of CS sourced from the STITCH database, which include cordycepin (Figure 2A), d‐mannitol (also known as cordycepic acid; Figure 2B), ergosterol (Figure 2C), β‐sitosterol (Figure 2D), adenosine (Figure 2E), and d‐glucoside (specifically cordyceps polysaccharide; Figure 2F). The figure also illustrates the intricate interactions between these ingredients and their respective target proteins. Cordycepin, as shown in Figure 2A, can activate CASP9, CASP8, CASP3, STAR, IL10, and FOXP3 and inhibit BCL7A, TLR4, and MYC. These proteins are involved in various cellular processes, such as apoptosis, cholesterol transfer, cytokine synthesis, immune regulation, and immune responses. The potential effects of cordycepin on cell apoptosis, inflammation inhibition, and immune regulation in COPD patients are highlighted based on the interactions with these target proteins.

d‐mannitol plays a significant role in various biological processes of COPD by activating AQP4, SRC, TGF‐β1, and NFAT5 and interacting with TLR4, NOS1, NOS2, and NOS3. Nitric oxide synthase (NOS) produces nitric oxide (NO), which is crucial for antimicrobial defence and antitumoral activities. SRC, a proto‐oncogene, is involved in multiple signalling pathways that regulate gene transcription, the immune response, cell adhesion, cell cycle progression, apoptosis, migration, and transformation.

Additionally, transforming growth factor beta 1 (TGF‐β1) modulates proliferation, differentiation, and other cellular functions in various cell types. Catalase (CAT) not only protects cells from hydrogen peroxide but also supports the growth of T cells and B cells. Ergosterol interacts with CYP51A1, FDFT1, MSMO1, OSBP, and OSBP2 and binds to OSBPL5, OSBPL9, OSBPL8, OSBPL10, and OSBPL11, affecting their functions related to oxidoreductase activity and oxysterol binding. β‐sitosterol can activate APOE and CASP3; catalyse DHCR24; inhibit SREBF2, SREBF1, ABCB11, and ICAM1; and interact with ABCG8, ABCG5, and CYP7A1. DHCR24 protects cells from oxidative stress by reducing CASP3 activity during apoptosis induced by oxidative stress. ICAM1 engagement promotes the assembly of endothelial apical cups during leukocyte transendothelial migration. The other proteins primarily mediate cholesterol and lipid metabolism.

Adenosine can activate ADORA1, ADORA2A, ADORA2B, and CXCR4; inhibit ADK and AHCY; and catalyse ADA, NT5E, and PNP (Figure 2E). These proteins primarily consist of adenosine kinase, receptors, deaminase, and phosphorylase. Adenosine deaminase (ADA) serves as a positive regulator of T‐cell coactivation by binding DPP4, which in turn regulates lymphocyte–epithelial cell adhesion. Chemokine (C‐X‐C motif) receptor 4 (CXCR4) transmits a signal by increasing intracellular calcium ion levels and enhancing mitogen‐activated protein kinase 1/3 (MAPK1/MAPK3) activation.

d‐glucoside (Figure 2F) was also predicted to interact with the LCT, GLB1, GALC, GALM, GLA, AKR1B1, AKR1B10, HMBS, and METTL7B proteins, which are associated mainly with glycometabolism and catalyse the hydrolysis of glycosphingolipids. AKR1B1 also has oxidoreductase and aldo‐keto reductase (NADP) activities. Unfortunately, there are no GEO data available for d‐mannitol, ergosterol, β‐sitosterol, adenosine, or d‐glucoside. Therefore, further analysis or verification of the predicted targets of CS ingredients from the STITCH database was not conducted.

**Figure 3:**
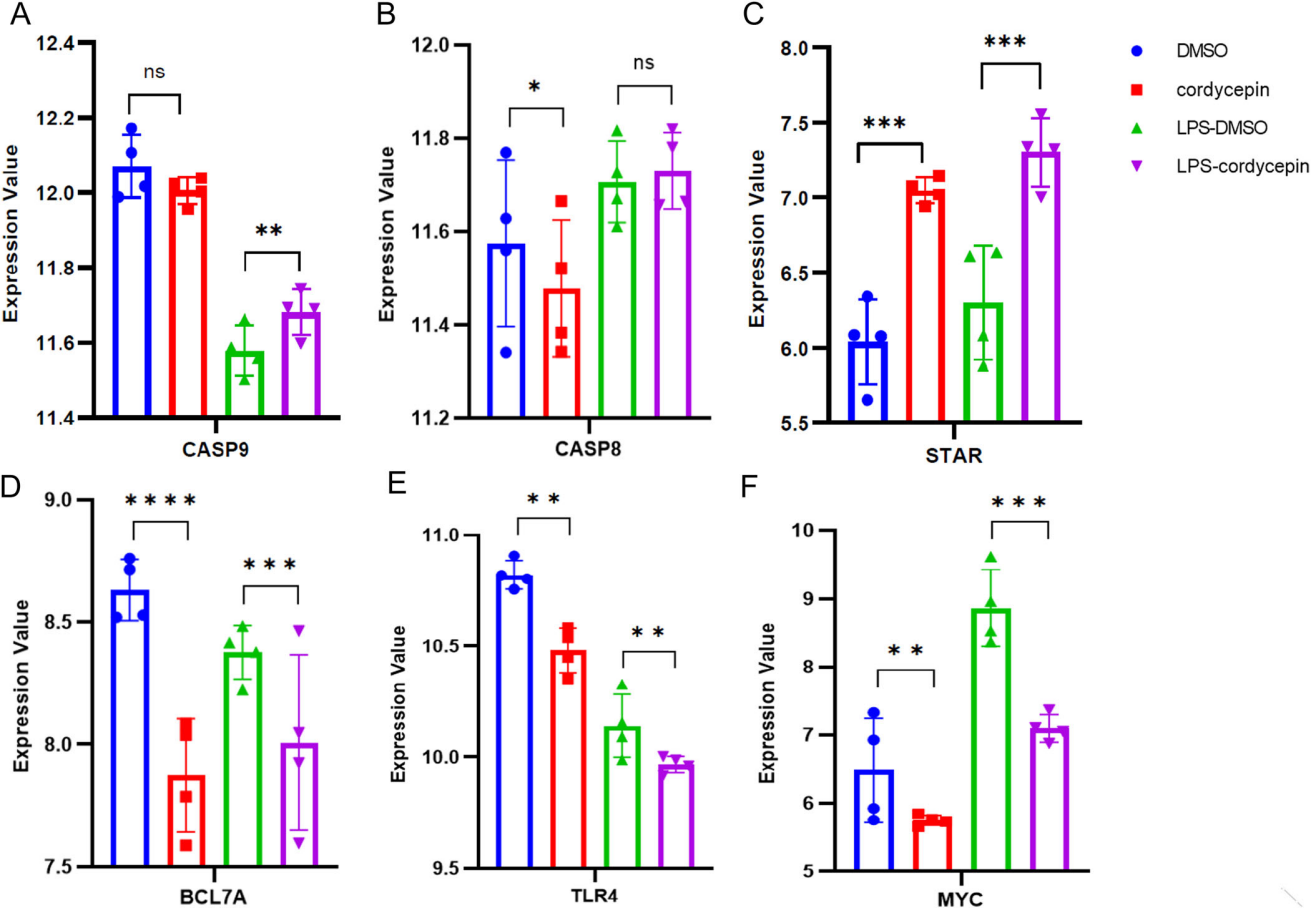
The verified effect of cordycepin on predicted protein by STITCH with GSE126157 data. RAW 264.7 cells were treated with cordycepin in the presence or absence of LPS. Cells were divided into four groups: DMSO control, cordycepin only, LPS‐treated with DMSO, and LPS‐treated with cordycepin. The figures show the fold change in gene expression compared to the control group, with statistical significance indicated by asterisks. (A) the deferential expression of gene CASP9; (B) the deferential expression of gene CASP8; (C) the deferential expression of gene STAR; (D) the deferential expression of gene BCL7A; (E) the deferential expression of gene TLR4; (F) the deferential expression of gene MYC. *p*‐value > 0.05 (ns), *p*‐value < 0.05 (*), *p*‐value < 0.01 (**), *p*‐value < 0.001 (***), *p*‐value < 0.0001 (****).

Figure 3 shows the interactions between cordycepin and the corresponding target proteins with the GSE126157 data, which were generated via GraphPad Prism 9.0 software. To investigate the anti‐inflammatory properties of cordycepin, RAW 264.7 cells were treated with cordycepin with or without bacterial lipopolysaccharide (LPS). RAW 264.7 cells were divided into four groups: group 1 was treated with only DMSO; group 2 was treated with only cordycepin; and group 3 cells were pretreated for 1 h with DMSO (group 1) or cordycepin (group 2), after which they were not treated (group 1 and group 2, DMSO and cordycepin) or treated with LPS (group 3 or group 4, LPS‐DMSO or LPS‐cordycepin). Regardless of LPS treatment, the cells were incubated for an additional hour [12].

Figure 3A shows there is no significant differential expression of the CASP9 gene (*p* value > 0.05 (ns)) between the DMSO‐treated and cordycepin‐treated groups, whereas there is a significant difference (*p* value < 0.01 (**)) between the LPS‐DMSO‐treated and LPS‐cordycepin‐treated groups; therefore, CASP9 gene expression is sensitive to cordycepin in LPS‐treated RAW 264.7 cells. Figure 3B shows the differential expression of the CASP8 gene (*p* value < 0.05 (*)) between the DMSO and cordycepin groups; therefore, CASP8 gene expression is sensitive to cordycepin in LPS‐untreated RAW 264.7 cells. Figure 3C shows the differential expression of the STAR gene (*p* value < 0.001 (***)) between DMSO and cordycepin in either the absence or presence of LPS. Figure 3D shows the differential expression of the BCL7A gene (*p* value < 0.0001 (****)) between the DMSO and cordycepin groups and between the LPS‐DMSO and LPS‐cordycepin groups (*p* value < 0.001 (***)). Figure 3E–F are similar.

As shown in Figure 3, cordycepin activated the expression of the STAR and CASP9 proteins; inhibited the expression of the BCL7A, TLR4, and MYC proteins; and had a minor effect on the expression of the CASP8 protein. On the basis of this information, we predicted that cordycepin may activate cell apoptosis, regulate inflammatory and immune responses, etc.

### 3.3 Analyses of the CS‐COPD Ingredient–Gene–Disease Network

**Figure 4:**
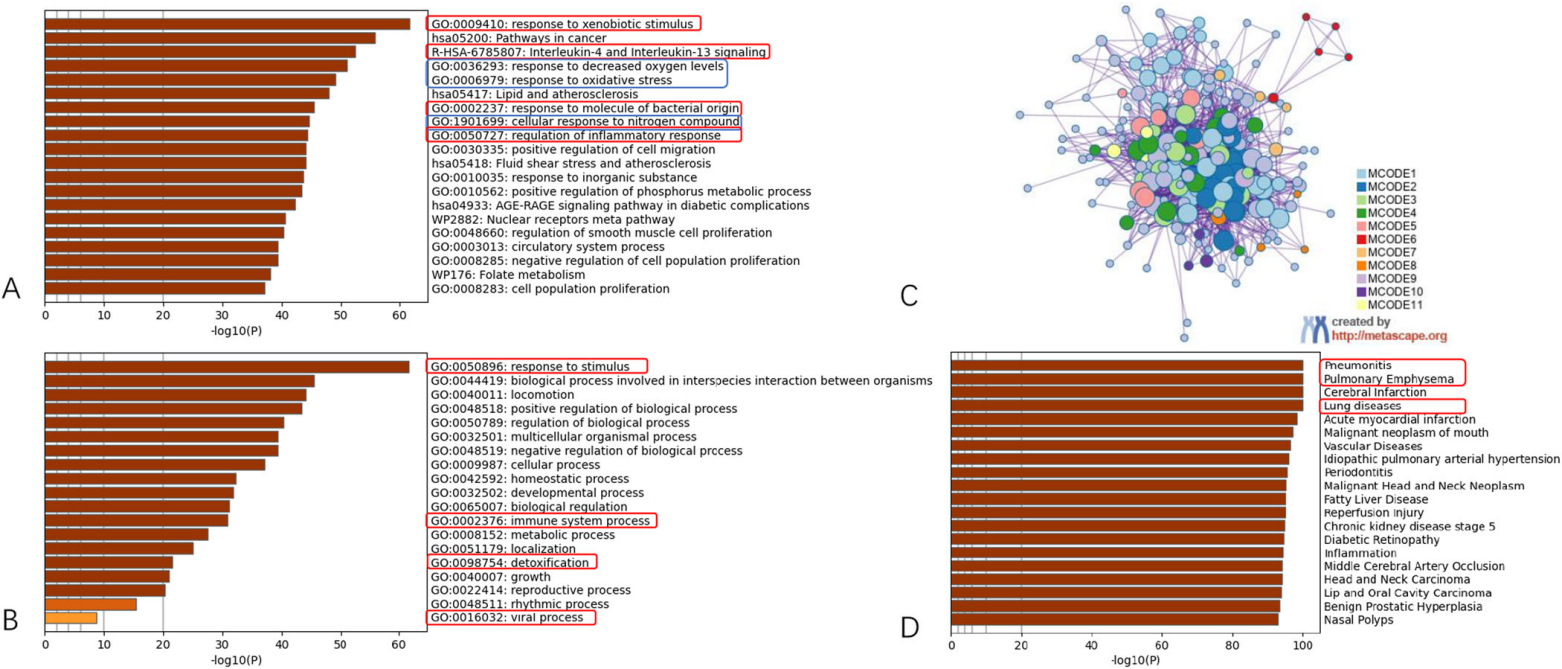
Analysis results on DEGs gene list by Metascape. (A) A bar graph illustrating the enriched terms in different input gene lists, with each bar color‐coded based on its corresponding *p*‐value. (B) This section offers a detailed summary of the primary GO biological processes. (C) The protein‐protein interaction network is visualized, emphasizing the MCODE components identified within the gene lists. (D) This section provides a concise summary of the enrichment analysis carried out in DisGeNET.

Figure 4 shows an in‐depth analysis of the DEGs via Metascape. Specifically, Figure 4A shows a bar graph highlighting enriched GO terms across various input gene lists. Each bar is colour‐coded according to its corresponding *p* value, encompassing inflammatory reactions such as response to xenobiotic stimulus, interleukin‐4 and interleukin‐13 signalling, response to molecules of bacterial origin, and regulation of the inflammatory response.

Furthermore, anti‐oxygen reactions, including response to decreased oxygen levels, response to oxidative stress, and cellular response to nitrogen compounds, are captured. Figure 4B provides a concise overview of the key GO biological processes. Leveraging the Medscape database, we conducted protein–protein interaction enrichment analysis on the 211 co‐targeted genes, and the resulting MCODE networks are visualized in Figure 4C. Last, Figure 4D focuses on enrichment analysis in DisGeNET, specifically targeting diseases or syndromes related to COPD, such as pneumonitis, pulmonary emphysema, cerebral infarction, lung diseases, and inflammation.

### 3.4 Verification of the Functions of CS and Cordycepin With GEO Data

GSE126157 comprehensively examined the influence of cordycepin on murine macrophage‐like cells under conditions with and without an inflammatory stimulus. Table 4 concisely outlines the DEGs in these cells analysed by GEO2R, with and without the presence of an inflammatory stimulus, pinpointing the top 10 genes. Notably, the predicted genes PRKAA2, MMP2, MMP9, UCP1, ADORA1, and ADORA3 were found to be nondifferentially expressed in this particular study.

**Table 4:** The top 10 DEGs in murine macrophage‐like cells treated with cordycepin, as determined by GEO2R analysis of GSE126157 data and potential relevance to the anti‐inflammatory effects of cordycepin.

Gene_Symbol	Gene_Name	*p*‐value	Adj.*p*. Val	*F*‐value
Ptgs2	Prostaglandin‐endoperoxide synthase 2	5.56e^−13^	1.42e^−10^	234.4876
Eif2s1	Eukaryotic translation initiation factor 2, subunit 1 alpha	6.17e^−12^	8.50e^−10^	169.6204
Jun	Jun oncogene	4.56e^−11^	3.99e^−09^	129.266
Gsk3b	Glycogen synthase kinase 3 beta	5.33e^−09^	1.89e^−07^	66.75285
Nfe2l2	Nuclear factor, erythroid derived 2, like 2	9.73e^−07^	1.48e^−05^	31.05232
Cebpb	CCAAT/enhancer binding protein (C/EBP), beta	1.48e^−06^	2.13e^−05^	29.10347
Akt2	Thymoma viral proto‐oncogene 2	6.19e^−04^	3.77e^−03^	10.20283
Pparg	Peroxisome proliferator activated receptor gamma	2.38e^−03^	1.18e^−02^	7.65267
Mapk14	Mitogen‐activated protein kinase 14	2.78e^−03^	1.34e^−02^	7.3875
Mapk1	Mitogen‐activated protein kinase 1	8.01e^−03^	3.19e^−02^	5.70702

*Note: p*‐value < 0.01; Adj.*p*.Val< 0.01; *F*‐value > 5.

Table 5 presents the results of the GO enrichment analysis of the effects of cordycepin on murine macrophage‐like cells via the STRING database. The primary GO terms highlighted are related to apoptotic processes, immune responses, and inflammatory signalling pathways. Table 6 shows the results of the KEGG enrichment analysis of the effects of cordycepin on murine macrophage‐like cells via the STRING database, emphasizing its involvement in apoptosis, virus infection‐related inflammatory diseases, and the IL‐17 and nuclear factor‐κB (NF‐κB) signalling pathways. Inflammation is a multifaceted response involving various cells and signalling molecules, such as the cytokine IL‐17, which is secreted by activated T cells.

**Table 5:** The GO enrichment of cordycepin on murine macrophage‐like cells by STRING database, identifies biological processes that are significantly enriched among the DEGs, including apoptotic, innate immune response, NF‐κB signalling pathways, etc.

Gene Set	Description	ES	NES	*p*‐value	FDR
GO:2001233	Regulation of apoptotic signaling pathway	0.59455	2.4439	< 2.2e^−16^	0.013661
GO:0097191	Extrinsic apoptotic signaling pathway	0.60933	1.9393	0.005141	0.20947
GO:0050867	Positive regulation of cell activation	0.56061	1.8175	0.013661	0.21959
GO:0045088	Regulation of innate immune response	0.57419	1.8406	0.01462	0.2292
GO:0007249	Canonical NF‐kappaB signal transduction	0.55043	1.7751	0.015504	0.23766
GO:0034612	Response to tumor necrosis factor	0.63564	1.8636	0.010076	0.24856

*Note:* ES, enrichment score; NES, normalize enrichment score; FDR, false discovery rate.

ES > 0.5; | NES | > 1; *p*‐value < 0.05; FDR < 0.25.

**Table 6:** The KEGG enrichment of cordycepin on murine macrophage‐like cells by STRING database, identifies signaling pathways that are significantly enriched among the DEGs, including apoptosis, IL‐17 and NF‐κB signalling pathways.

KEGG pathway	Description	Strength	FDR
mmu04210	Apoptosis	0.73	0.0196
mmu04625	C‐type lectin receptor signaling pathway	0.82	0.0151
mmu05210	Colorectal cancer	0.78	0.0372
mmu05169	Epstein‐Barr virus infection	0.63	0.0196
mmu05161	Hepatitis B	0.71	0.0196
mmu05163	Human cytomegalovirus infection	0.59	0.0204
mmu04657	IL‐17 signaling pathway	0.96	0.00076
mmu05321	Inflammatory bowel disease	0.93	0.0196
mmu04931	Insulin resistance	0.75	0.0246
mmu04064	NF‐kappa B signaling pathway	0.79	0.0196

*Note:* Strength > 0.50; FDR < 0.25.

Upon stimulation by IL‐17, fibroblasts and other cells are prompted to secrete inflammatory and haematopoietic cytokines, including IL‐6, IL‐8, G‐CSF, and stem cell factor (SCF). The IL‐17 family transmits signals through their respective receptors, activating downstream pathways such as the NF‐κB, MAPK, and C/EBP pathways and ultimately inducing the expression of antimicrobial peptides, cytokines, and chemokines. Additionally, miR‐31 exacerbates inflammation and apoptosis in COPD rats by activating the NF‐κB signalling pathway.

**Figure 5:**
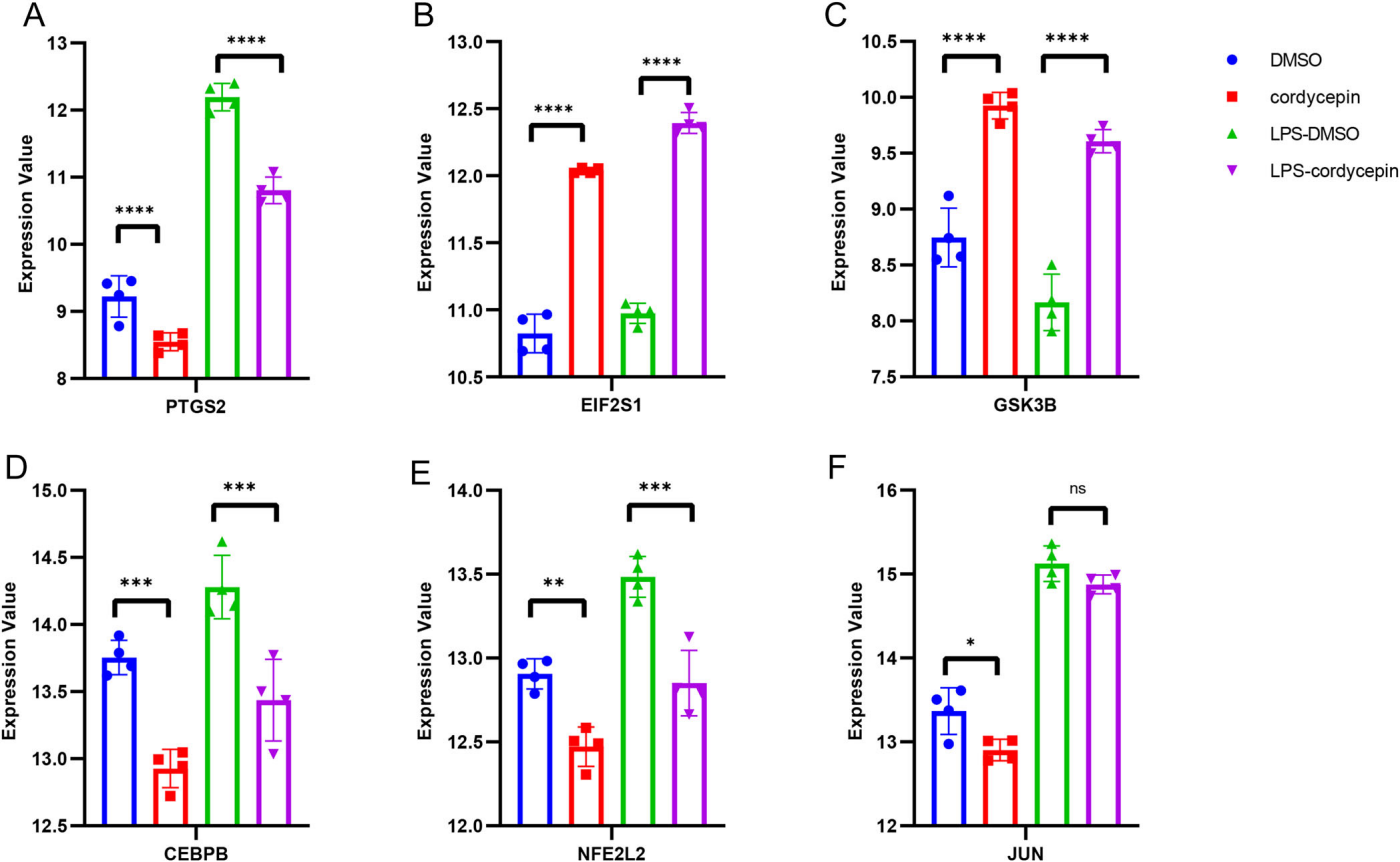
The effect of cordycepin on murine macrophage‐like cells with CS‐targeted DEGs. This figure demonstrates the effect of cordycepin on the expression of selected DEGs in murine macrophage‐like cells, as identified through transcriptomic analysis (GSE126157). A two‐way ANOVA was used to compare the effects of cordycepin treatment (with or without LPS) on gene expression levels, with statistical significance indicated by asterisks. (A) the deferential expression of gene PTGS2; (B) the deferential expression of gene EIF2S1; (C) the deferential expression of gene GSK3B; (D) the deferential expression of gene CEBPB; (E) the deferential expression of gene NFE2L2; (F) the deferential expression of gene JUN. *p*‐value > 0.05 (ns), *p*‐value < 0.05 (*), *p*‐value < 0.01 (**), *p*‐value < 0.001 (***), *p*‐value < 0.0001 (****).

Figure 5 shows the profound influence of cordycepin on murine macrophage‐like cells, highlighting the cordycepin‐specific DEGs (top 6), via a rigorous two‐way ANOVA comparing DMSO vs. cordycepin and LPS‐DMSO vs. LPS‐cordycepin. This comprehensive graphical portrayal was created via advanced GraphPad Prism 9.0 software. Figure 5 shows a similar content to that shown in Figure 3. This figure clearly demonstrates the capacity of cordycepin to stimulate the expression of the EIF2S1 and GSK3B proteins while simultaneously suppressing the expression of the PTGS2, CEBP8, NFE2L2, and JUN proteins. Figure 5 verifies the information in Table 4.

**Figure 6:**
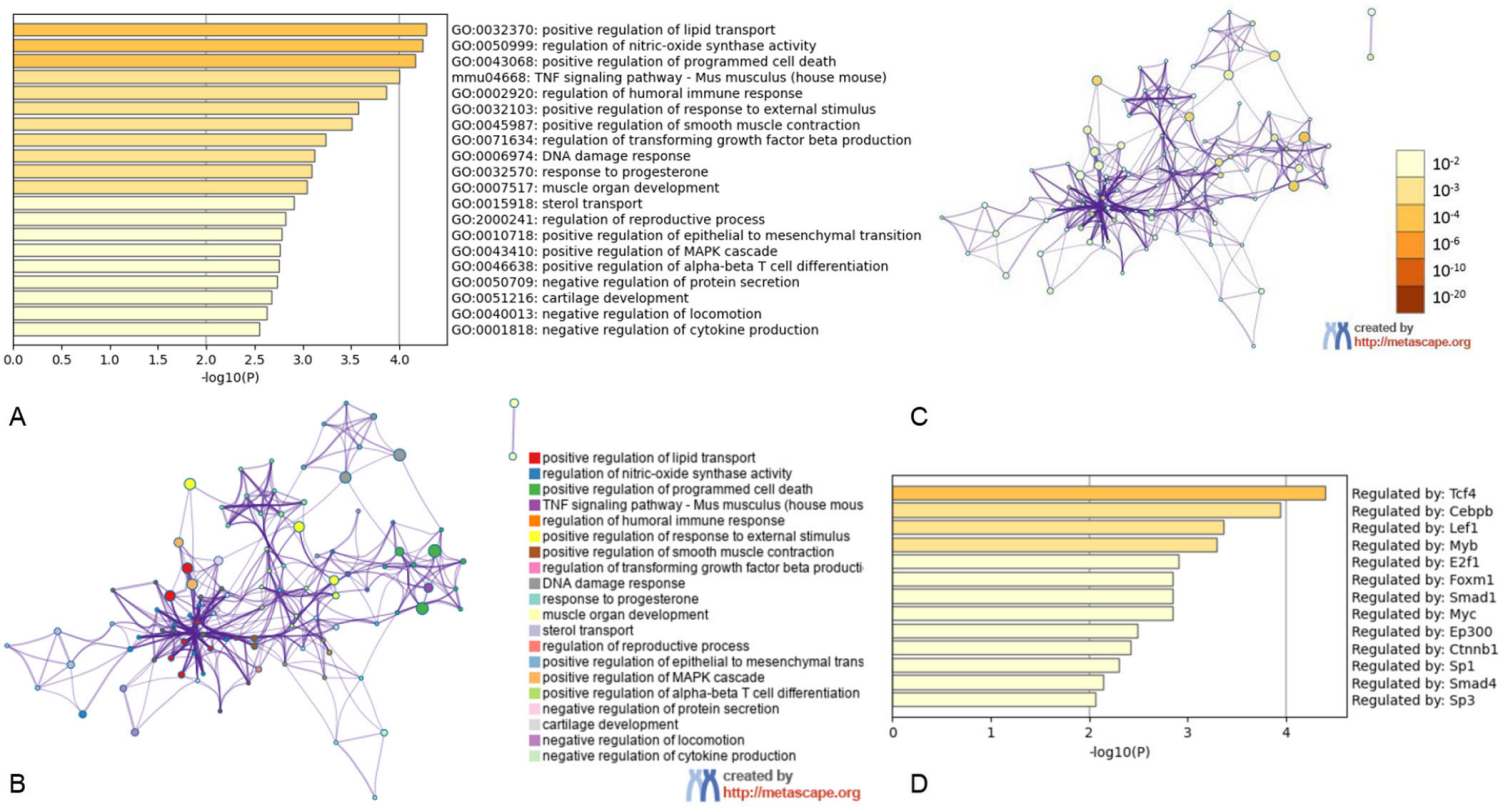
Metascape visualizes the DEGs clusters of LPS treated RAW 264.7 cell lines. (A) Bar graph of enriched terms across input gene lists, colored by *p*‐value (top 20). Network of enriched terms: (B) colored by cluster ID, where nodes that share the same cluster ID are typically close to each other; (C) colored by *p*‐value, where terms containing more genes tend to have a more significant *p*‐value. (D) Summary of enrichment analysis in TRRUST.

Figure 6 provides a visual representation of the clusters of DEGs in the LPS‐treated RAW 264.7 cell line, thereby validating the predicted effects of cordycepin treatment on COPD. Specifically, Figure 6A shows a bar graph depicting the enriched terms derived from the input gene lists, with each colour representing the respective *p* value (top 20 terms).

The network of these enriched terms, depicted in Figure 6B, is coloured according to cluster ID, indicating that nodes sharing the same ID are often in close proximity. Additionally, Figure 6C, coloured by *p* value, highlights that terms with a greater number of genes tend to have a more significant *p* value. Finally, Figure 6D shows a summary of the enrichment analysis conducted at TRRUST.

### 3.5 Molecular Docking Analysis Results of CS Ingredients and COPD‐Co‐Targeted Proteins

**Figure 7:**
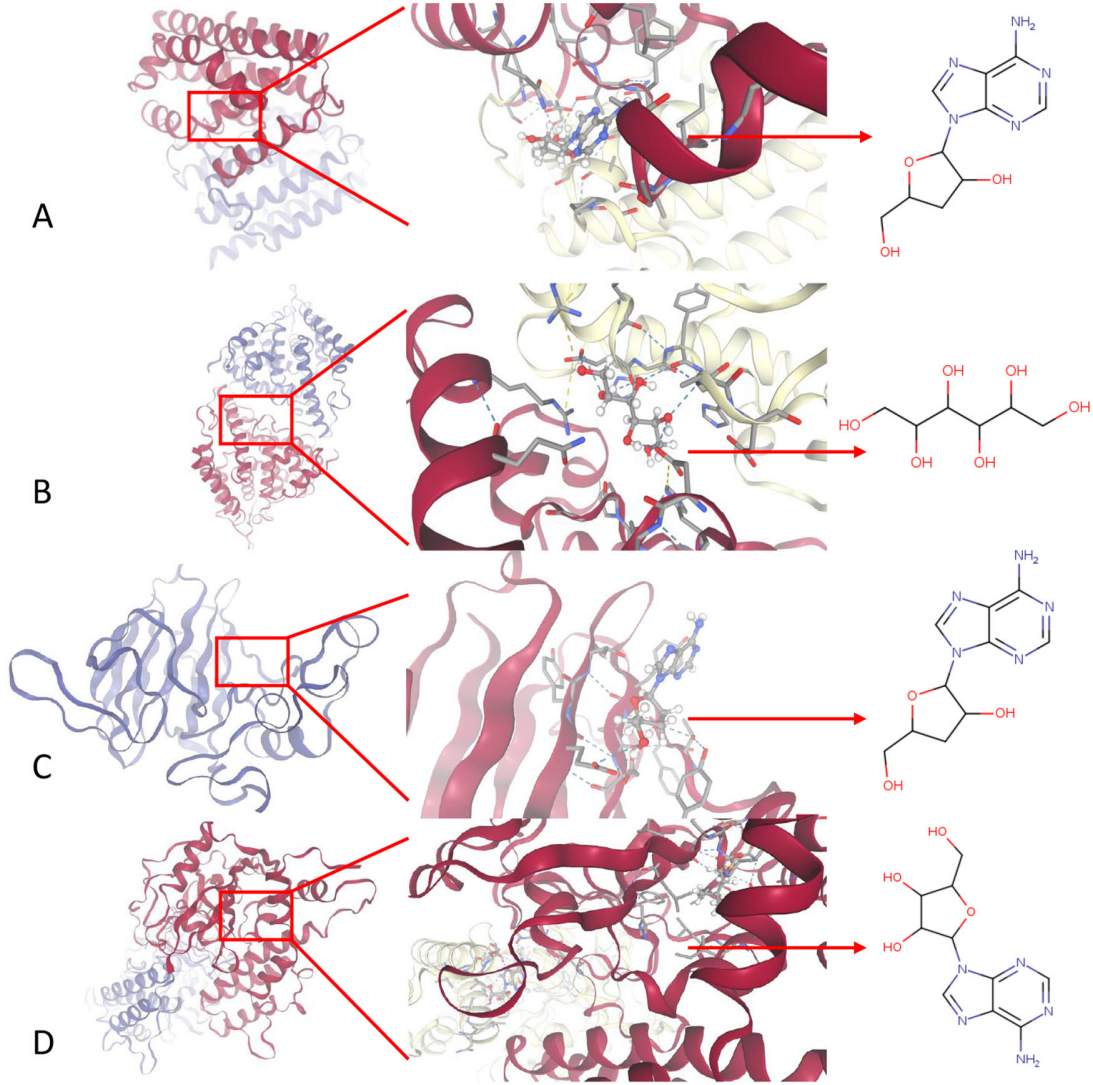
Molecular docking results for CS ingredients and COPD co‐targeted proteins. (A) Binding mode of Cordycepin to HMOX1(1N3U), AC Score = 26.22; SwissParam Score = −6.9978; (B) Binding mode of d‐mannitol to PDE4A(3TVX), AC Score = 46.50; SwissParam Score = −6.0003; (C) Binding mode of Cordycepin to JUN(7P20), AC Score = 28.08; SwissParam Score = −6.9978; (D) Binding mode of adenosine to PARP1(7KK2), AC Score = 40.19; SwissParam Score = −6.8633.

We conducted molecular docking analysis with SwissDock to evaluate the affinity of the CS ingredients for their intended COPD co‐targets. The binding poses and interactions of two CS ingredients with four proteins were determined, along with the generation of the binding energy for each interaction (Figure 7). Our findings revealed that each CS ingredient bound to its respective targeted protein through clearly observable hydrogen bonds and robust electrostatic interactions.

## 4 Discussion

Inhalation of toxic substances such as cigarette smoke or PM2.5 leads to lung oxidative stress, airway inflammation, cytokine release, and immune cell activation, ultimately leading to cell apoptosis and the progression of enlarged alveolar spaces during the development of COPD. Although TCM has demonstrated its anti‐COPD effects in clinical therapy with many patient drugs made from CS or its ingredients, the underlying mechanisms and specific roles in COPD still require further elucidation.

### 4.1 The Main Effects of CS Ingredients in the Treatment of COPD in This Study

This study revealed that CS may have therapeutic effects on COPD through anti‐inflammatory, antiapoptotic, antioxidant, and immune‐regulating effects on various proteins and signalling pathways, including the anti‐inflammatory signalling pathway, such as response to xenobiotic stimulus, interleukin‐4 and interleukin‐13 signalling, response to molecules of bacterial origin, and regulation of the inflammatory response; the apoptotic signalling pathway, such as the regulation of the apoptotic signalling pathway and extrinsic apoptotic signalling pathway, which are involved in the apoptotic signalling pathway; and the antioxidative signalling pathway, including response to decreased oxygen levels, response to oxidative stress, and cellular response to nitrogen compounds.

We conclude that CS treatment of COPD is associated with anti‐inflammatory, antiapoptotic, and antioxidant effects. Unique genes regulated by CS treatment are significantly involved in the regulation of pathways related to ECM‐receptor interactions and the haematopoietic cell lineage in different stages of dendritic cell physiology under CS treatment [18].

In this study, CS was predicted to affect COPD by targeting genes such as PARP1, PDE4A, PDE4B, PDE4C, PDE4D, PTGDR2 (*p* = 4.61e^‐03^, TTD), HMOX1, and MMP1 (*p* = 3.65e^‐02^, OMIM). Cordycepin utilizes HMOX1, PDE4B, and PDE4D as targeted gene therapy strategies for COPD treatment. d‐mannitol specifically targets PDE4A, PDE4B, PDE4C, and PDE4D for gene‐based treatment of COPD. Ergosterol utilizes PDE4B and PDE4D, and adenosine targets PARP1, PTGDR2, PDE4A, PDE4B, PDE4C, PDE4D, and HMOX1 as co‐targeted proteins in the therapeutic approach for COPD.

### 4.2 Published Data on the Roles of CS Ingredients in the Treatment of COPD

Dong et al. examined the chemical composition of cultivated CS powder and highlighted the presence of nucleosides, sterols, and polysaccharides. Specifically, nucleosides, ergosterol, and polysaccharides in cultivated CS were found to possess antifibrotic properties [19].

Cordycepin, a purine nucleoside antimetabolite and antibiotic, has shown promise in various therapeutic applications, such as antitumour, antioxidant, and anti‐inflammatory effects. Several studies have demonstrated that cordycepin can reduce inflammation and pain in animal models, with potential anti‐asthmatic effects attributed to its ability to inhibit inflammation and protect against lung injuries. Furthermore, cordycepin exhibits strong anti‐inflammatory activity in mouse models of acute lung injury and asthma by mitigating the inflammatory response and oxidative stress [20]. Moreover, cordycepin downregulates ICAM‐1, IL‐4, IL‐5, and IL‐13 while also inhibiting the p38‐MAPK and NF‐κB signalling pathways. Additionally, cordycepin significantly inhibits LPS‐induced lung inflammation, as evidenced by the reduced lung wet/dry ratio, inflammatory cytokines (TNF‐α and IL‐1β), NF‐κB activation, and oxidative stress. This inhibition is achieved through activation of the Nrf2 signalling pathway in a dose‐dependent manner [21, 22].

The MAPK family, particularly p38‐MAPK, is central to COPD pathogenesis by regulating inflammation, cellular senescence, and tissue remodeling. Recent evidence confirms MAPK's role in immune dysregulation during pulmonary inflammation [23]. COPD‐associated inflammation is driven by TNF‐α, IL‐1β, IL‐6, and IL‐8 (CXCL8) released from epithelial cells, macrophages, and neutrophils. These cytokines amplify lung injury and correlate with disease severity [24].

Ergosterol effectively mitigates inflammatory responses, oxidative stress, and apoptosis in COPD through the NF‐κB/p65 pathway in both 16HBE cells and BALB/c mice [25]. It restores superoxide dismutase (SOD) activity; suppresses pro‐inflammatory cytokines, such as tumour necrosis factor‐α (TNF‐α), interleukin‐6 (IL‐6), and interleukin‐1β (IL‐1β); and modulates the protein expression of the JAK3/STAT3/NF‐κB pathway in mice [26].

In RAW264.7 cells and SD rats, ergosterol treatment reduced CSE‐induced inflammation by decreasing the levels of ROS, IL‐6, and TNF‐α while increasing the levels of IL‐10 and TGF‐β, leading to a shift in macrophage polarization from M1 to M2 both in vitro and in vivo [27]. Additionally, β‐sitosterol downregulated the expression of HIF‐1α, AKT1, JUN, and RELA in A549 cells [28].

Cordyceps polysaccharides, comprising mannose, adenosine, galactose, arabinose, xylose, glucose, and fucose, exhibit diverse biological activities, including immune regulation, antitumour effects, antiaging properties, antifatigue benefits, and regulation of lipid and sugar metabolism. These polysaccharides can mitigate oxidative stress and activate the MyD88/NF‐κB signalling pathway [29, 30].

### 4.3 CS Targets Inflammation‐Related Factors and Pathways in the Treatment of COPD

CS has been shown to reduce the levels of IL‐8, TNF‐α, TGF‐β1, MDA, Th17/Treg, Th17, and CD8 + ; increase CD3 + , CD4 + , CD4 + /CD8 + , and SOD levels; and regulate the PI3K/Akt and CAMP signalling pathways to alleviate symptoms of COPD [31]. CS treatment has been shown to significantly improve airway wall thickening, collagen deposition, airway wall fibrosis, smooth muscle hypertrophy, and epithelial hyperplasia in rat models of COPD. Furthermore, CS administration has been linked to reduced inflammatory cell accumulation and decreased production of the inflammatory cytokines TNF‐α, IL‐8, and TGF‐β1 in rats with COPD.

Biochemical and histological signs of airway remodelling, such as collagen I and α‐SMA levels, were notably decreased in CS‐treated rats. Moreover, CS treatment decreased the expression of TGF‐β1, TβR I, and TβR II while increasing Smad7 expression in rats with COPD. These findings suggest that CS treatment may be a beneficial therapeutic approach for COPD [32]. Additionally, CSE (cigarette smoke extract) has been found to induce senescence in human bronchial epithelial cells, with the ROS/PI3K/AKT/mTOR signalling pathway potentially playing a key role in this process. Notably, CS has been shown to inhibit CSE‐induced senescence [33].

In rat models of COPD, the expression levels of IFN‐γ, TNF‐α, and IL‐12 p70 proteins are higher in the CS‐treated rats compared to both the COPD rat models and wild‐type rats. The CS may act on dendritic cells in the COPD rat models, resulting in the production of Th1 cytokines, including IL‐12 p70, and promoting the production of IFN‐γ and TNF‐α by T cells [34].

In another study, C57 mice were subjected to LPS and smoke exposure and subsequently treated with fresh CS extraction for a period of 13 weeks to establish a COPD mouse model with CS intervention. The findings revealed a notable downregulation of IL‐6, IL‐8, and IL‐1β (*p* < 0.05) in the CS‐treated COPD mouse model, accompanied by an upregulation of IFN‐γ, in comparison to both wild‐type mice and the untreated COPD mouse model [35].

### 4.4 CS Targets Cellular Apoptosis and Autophagy in the Treatment of COPD

As reported, compared with the CSE treated COPD A549 cell model, the cell apoptosis rate in CS treated group was significantly reduced (*p* < 0.01). Additionally, there were fewer instances of chromatin agglutination, nuclear atrophy, and apoptotic bodies observed. The expressions of p‐PERK, p‐eIF2α, and Bcl‐2 were downregulated in the CS treated group, whereas the expression of Bax was upregulated (*p* < 0.01) [36]. Ergosterol, the main ingredient of CS, suppressed apoptosis by inhibiting the expression of the apoptosis‐related proteins both in vitro and in vivo [25].

Another main ingredient of CS, Cordycepin inhibited cell growth by inducing apoptosis and autophagy in human NSCLC cells with downregulation of c‐FLIPL which suppresses the activity of caspase‐8. Cordycepin stimulated autophagy through suppressing mTOR signalling pathway [37]. Cordycepin inhibits the ERK/Slug signalling pathway through the activation of GSK3β which, in turn, upregulates Bax, and leading to apoptosis of the lung cancer cells [38]. Cordycepin inhibits the expression of apoptosis‐related proteins via the mitochondrial pathway, and induces apoptosis by activating caspase‐3, caspase‐9, and cytochrome C [39].

Oxidative stress in COPD stimulates autophagy via the upregulation of p62/SQSTM1 mediated by NF‐κB. Additionally, autophagy plays a pivotal role in the selective degradation of damaged and dysfunctional mitochondria, known as mitophagy [40].

### 4.5 CS Targets Oxidative Stress in the Treatment of COPD

Oxidative stress is a key factor in the development and progression of COPD and results in frailty and negative outcomes. Disruption of the balance between antioxidants and oxidants initiates pathological processes in COPD, with ROS playing a crucial role in tissue and cell damage [41]. CS targeting of oxidative stress for the treatment of COPD can be effectively utilized to alleviate symptoms and improve the quality of life of patients suffering from this chronic respiratory disease.

CS encompasses a diverse array of polyphenolic ingredients, flavonoids, alkaloids, superoxide dismutases (SODs), and other potent active ingredients renowned for their robust antioxidant properties. These active ingredients aid in combating free radicals within the lungs, subsequently mitigating oxidative damage to tissues and cells and delaying the progression of COPD. This sequence involves the activation of alveolar macrophages, cell death, and the release of inflammatory cytokines such as JNK, NF‐κB, IL‐1, IL‐6, IL‐8, IL‐1β, and TNF‐α. Moreover, oxidative stress‐induced dysfunction of mitochondria contributes to inflammation and airway remodelling, ultimately resulting in dysfunction of the airway epithelium and abnormalities related to COPD [42].

Clinical and preclinical studies have demonstrated that antioxidants in CS can reduce ROS levels and regulate the expression of pro‐inflammatory genes [43]. The oxidative balance score (OBS) is commonly used to assess oxidative stress related to diet and lifestyle, and it has been found to be inversely correlated with frailty in COPD patients [44].

Several clinical studies and trials have investigated the efficacy of CS in respiratory diseases, with positive outcomes in terms of improved lung function, reduced exacerbations, and improved quality of life [45]. The total effective rate of the treatment group was 86.8%, while the total effective rate of the control group was 65.0%. The difference in efficacy between the two groups was statistically significant (*χ* ^2^ = 6.23, *p* > 0.05) [46]. CS demonstrates significant therapeutic potential in pulmonary fibrosis by specifically inhibiting mitochondrial complexes I and II. This dual targeting effectively attenuates mitochondrial ROS generation, ameliorates oxidative stress, and suppresses inflammatory cascades, collectively contributing to its anti‐fibrotic efficacy [47].

Multi‐omic data indicate that CS treatment enhances mitochondrial biogenesis and function in mice subjected to LPS‐induced S‐AKI. This includes increased expression of genes involved in mitochondrial DNA replication and transcription, as well as proteins critical for mitochondrial oxidative phosphorylation [48].

In LPS‐activated THP‐1 macrophages, CSE manifests robust anti‐inflammatory and antioxidant effects through Cytokine suppression, including downregulation of pro‐inflammatory mediators (IL‐6, TNF‐α, IL‐1β, IL‐8), activation of the Nrf2/HO‐1 antioxidant pathway, and modulation of Toll‐like receptor (TLR) and NOD‐like receptor (NLR) signaling networks [49].

Therefore, CS alleviates COPD symptoms means that CS treatment reduced sputum inflammatory cells (*p* < 0.01), decreased serum IL‐6 (*p* < 0.05), and improved forced expiratory volume in 1 s/forced vital capacity (FEV1/FVC) ratio (*p* < 0.01) compared to controls. CS has been reported to have minimal side effects, such as mild abdominal distension and throat discomfort. Studies in rats and rabbits have not revealed any significant toxic effects on haematological parameters. Additionally, CS has been shown to enhance lung function, exercise capacity, quality of life, and arterial oxygen levels in COPD patients.

## 5 Conclusions

CS represents a promising complementary therapy for COPD that targets key pathological mechanisms, such as inflammation, apoptosis, and oxidative stress. Its incorporation into COPD management could improve patient outcomes and quality of life. Future research should focus on elucidating the precise molecular mechanisms involved and conducting rigorous clinical trials to validate its efficacy and safety.

## Author Contributions

Z.Z. performed the project, data curation and writing the manuscript. Y.K. participated in data curation, formal analysis, discussion, and visualization. Q.K. designed the project, funding acquisition, reviewed and edited the manuscript, and provided comments and directions. All authors have read and agreed to the published version of the manuscript.

## Ethics Statement

The authors have nothing to report.

## Conflicts of Interest

The authors declare no conflicts of interest.

## Data Availability

The authors have nothing to report.
